# Bioresorbable Polylactic Acid Matrix for Chronic Non-Healing Wounds: First Clinical Experience in Europe

**DOI:** 10.3390/jpm16010010

**Published:** 2025-12-31

**Authors:** Ioannis-Fivos Megas, Paul Christian Fuchs, Florian Pinterits, Akshay Mrigendra Jain, Panagiotis Fikatas, Götz Habild, Sarina Delavari, David Breidung

**Affiliations:** 1 Department of Orthopaedic and Trauma Surgery, Center of Plastic Surgery, Hand Surgery and Microsurgery, Evangelisches Waldkrankenhaus Spandau, Stadtrandstraße 555, 13589 Berlin, Germany; fivos.megas@gmail.com (I.-F.M.); florian.pinterits@jsd.de (F.P.); akshay.jain@jsd.de (A.M.J.); goetz.habild@jsd.de (G.H.); 2 Department of Surgery, Evangelisches Krankenhaus Königin Elisabeth Herzberge, Herzbergstraße 79, 10365 Berlin, Germany; p.fikatas@keh-berlin.de; 3 Department of Plastic, Reconstructive, Hand and Burn Surgery, Merheim Medical Centre Cologne, University of Witten/Herdecke, 51109 Cologne, Germany; 4 Department of Health, University of Witten/Herdecke, 58455 Witten, Germany; 5 Department of Surgery, Campus Charité Mitte and Campus Virchow-Klinikum, Charité-Universitätsmedizin, Corporate Member of Freie Universität Berlin, Humboldt-Universität zu Berlin, Berlin Institute of Health, Augustenburger Platz 1, 13353 Berlin, Germany; 6 Department of Plastic, Reconstructive and Hand Surgery, Center for Severe Burn Injuries, Klinikum Nürnberg, Paracelsus Medical University, Breslauer Str. 201, 90471 Nuremberg, Germany; sarinadelavari@yahoo.de; 7 Department of Plastic Surgery and Hand Surgery, University Hospital Zurich, Rämistrasse 100, 8091 Zurich, Switzerland

**Keywords:** polylactic acid, chronic wounds, venous leg ulcer, wound healing, wound closure matrix

## Abstract

**Background/Objectives**: Bioresorbable polylactic acid (PLA) matrices have shown promise in supporting wound healing through their biocompatibility, tissue integration, and potential involvement in immune regulatory mechanisms. This study aimed to analyze the clinical performance of a PLA-based matrix in the treatment of chronic wounds under real-world conditions in a single-center setting. **Methods**: This retrospective study included patients with chronic wounds treated with the polylactic acid matrix at Evangelisches Waldkrankenhaus Spandau between February 2023 and February 2025. Wounds were surgically debrided in the operating room prior to matrix application. The matrix remained in place until resorption or detachment, with reapplications occurring at a mean interval of approximately 14 days. Data was anonymized and analyzed descriptively. **Results**: A total of 14 patients with 16 chronic wounds were treated in this study. The mean patient age was 76.1 years. The most common underlying causes were ischemia and trauma, with an average wound size of 23.6 cm^2^. Complete wound closure was achieved in 15 out of 16 cases (93.8%), with a mean time to complete wound closure of 72.9 days. The average duration of hospitalization was 24.8 days. **Conclusions**: The polylactic acid matrix demonstrated a high rate of short-term wound closure in a heterogeneous cohort of chronic wounds, with a mean time to closure of 73 days and no requirement for skin grafting. Further prospective studies with standardized long-term follow-up are warranted.

## 1. Introduction

Chronic wounds represent a growing challenge for healthcare systems worldwide. In the United States, approximately 6.5 million individuals are affected, with annual treatment costs exceeding 25 billion US dollars [1,2,3]. In Germany, epidemiological data indicate that nearly 800,000 individuals live with chronic wounds, with approximately 200,000 new cases occurring annually [4]. Demographic aging and the increasing prevalence of diabetes mellitus, peripheral arterial disease, and multimorbidity are expected to further increase incidence rates. Chronic wounds are associated with significant impairment in quality of life, increased morbidity, and elevated mortality [1,3].

The pathophysiology of chronic wounds is multifactorial and characterized by persistent inflammation, impaired angiogenesis, and dysregulated extracellular matrix remodeling. These mechanisms result in delayed granulation tissue formation and re-epithelialization, thereby increasing susceptibility to infection and prolonged wound persistence [2]. In recent years, bioresorbable synthetic wound matrices have been introduced as adjuncts to standard wound care. Among these, polylactic acid (PLA)–based matrices have gained attention due to their biocompatibility, predictable degradation profile, and capacity to support tissue integration. Originally applied in burn care, PLA matrices have demonstrated the ability to promote granulation tissue formation and provide temporary wound coverage in complex defects [5,6].

In clinical practice, chronic wounds often exhibit considerable interindividual heterogeneity in terms of etiology, wound morphology, tissue quality and underlying comorbidities, meaning that standardized treatment algorithms are often inadequate. As a result, effective wound management frequently requires individualized, patient-centered therapeutic strategies that can be adapted to the specific biological and clinical context of each wound. In this setting, advanced wound matrices may serve as flexible adjuncts within a personalized medicine approach to chronic wound care. Studies have furthermore shown that PLA matrices can modulate local immune responses, support granulation tissue formation, and exert both angiogenic and lymphangiogenic effects—an advantage particularly relevant in exudative or inflamed wound environments. The lactate released has been identified as a key initiator of these processes. It promotes angiogenesis and accelerates wound healing through HIF-1α-mediated signaling pathways and redox-dependent mechanisms—even under normoxic conditions [7,8,9]. In addition, regenerative scaffolds enriched with adipose-derived mesenchymal stem cells have demonstrated enhanced (lymph-)angiogenic activity, thereby contributing to tissue regeneration in complex wound scenarios [10].

Recent clinical data further support the use of polylactic acid–based matrices in complex wound settings [11,12,13]. A recently published case report described favorable clinical observations using a PLA-based matrix, highlighting its biocompatibility, integration into the wound bed, and suitability for challenging wound environments [11]. These findings contribute to the growing interest in synthetic bioresorbable matrices as adjuncts to standard wound care and highlight their potential relevance in individualized, patient-centered wound management within the framework of personalized medicine. A multicenter retrospective study by Liden et al. demonstrated the clinical efficacy of a polylactide-based wound matrix in both acute and chronic wounds of various etiologies, reporting accelerated healing and reduced surgical intervention rates [14]. However, there remains a paucity of single-center data on the real-world use of this technology.

The aim of this single-center retrospective observational case series is to present our initial experience with a bioresorbable polylactic acid (PLA) matrix in the treatment of chronic and therapy-resistant wounds of diverse etiologies. Special attention is given to the matrix’s ability to integrate into the wound bed and its potential contribution to improving the likelihood of wound closure. To the best of our knowledge, this is the first study in Europe to investigate the use of a polylactide-based matrix specifically in the context of chronic wounds.

## 2. Materials and Methods

### 2.1. Study Design and Setting

This retrospective observational study was conducted at the Department of Orthopaedic and Trauma Surgery, Center of Plastic Surgery, Hand Surgery and Microsurgery at Evangelisches Waldkrankenhaus Spandau in Berlin, Germany. It included patients treated between February 2023 and February 2025. All patients with chronic wounds of defined etiologies (ischemic, infectious, post-traumatic, and iatrogenic/postoperative) were treated as part of routine clinical care with a synthetic polylactic acid matrix (SupraSDRM Biodegradable Matrix Wound Dressing, PolyMedics Innovations GmbH, Denkendorf, Germany), a CE-marked skin substitute approved for clinical use in Europe.

### 2.2. Patient Population and Clinical Management

In this study, a chronic wound was defined as a wound that failed to demonstrate meaningful clinical healing, characterized by a lack of progressive reduction in wound size or sustained epithelialization, after at least four weeks of appropriate standard wound care, in accordance with commonly accepted clinical definitions. All 16 wounds included in this analysis fulfilled the criterion of chronicity prior to the first application of the polylactic acid (PLA) matrix. Only adult patients aged 18 years or older were eligible for inclusion. Apart from the predefined age restriction, all consecutive patients treated with PLA matrix during the study period were included without further selection. Standard wound care comprised regular wound cleansing, debridement if necessary, infection control measures, pressure offloading or compression therapy when appropriate. The optimization of underlying comorbidities, including diabetes mellitus, smoking status, peripheral arterial disease, and relevant cardiovascular conditions was also part of routine care. Offloading and compression therapy were applied according to established international guideline-based standards and indication-driven clinical protocols. Systemic antibiotic therapy was initiated only in the presence of clinical signs of wound infection, including local inflammatory changes, periwound reaction, or developing phlegmon. Perioperative management followed institutional standards, including administration of a single-shot antibiotic prophylaxis. Exclusion criteria comprised malignancy, critical limb ischemia, and severe acute wound infection (e.g., extensive phlegmon or necrotizing fasciitis).

### 2.3. Intervention and Follow-Up

Prior to the initial application, all wounds underwent tangential surgical debridement in the operating room under sterile conditions to remove potential necrotic tissue and biofilm. A viable wound bed was confirmed via the appearance of a bleeding response and the absence of devitalized tissue. Immediately afterwards, the polylactic acid matrix was then applied directly to the wound surface and covered with a suitable secondary dressing. Depending on individual wound characteristics—such as exudate levels, depth, and surface area—additional materials such as silicone mesh, absorbent gauze, or antimicrobial dressings were used as needed. The dressing was secured using conventional bandaging techniques.

The matrix was not changed on a fixed schedule but remained in place until it had either fully resorbed or detached. The average interval between applications was 13.7 ± 2.0 days (range: 8.0–15.8 days). The mean number of applications per wound was 5.3 ± 2.9 (range: 2–13). Reapplication was performed after wound cleansing, involving the application of a secondary dressing, and was guided by either visible degradation of the matrix or clinical judgment regarding its integration. Infections were diagnosed primarily based on clinical signs, including erythema, warmth, swelling, pain, and purulent exudate. Microbiological swabs were obtained selectively in cases with suspected infection; routine cultures were not performed in all patients.

Patients were routinely observed as part of standard clinical care. Follow-up was not protocol-driven but based on regular outpatient visits until wound closure. After discharge, patients were monitored with weekly outpatient visits, during which the progression of wound healing, wound size, and signs of complications were assessed. Unscheduled visits were arranged as needed.

In cases of suspected local infection or relevant bacterial colonization during the treatment course, the polylactic acid matrix was removed, and wound care was temporarily switched to topical antimicrobial management, including antiseptic wound irrigation and antimicrobial dressings. Additional visits after wound closure were only scheduled in cases of suspected or manifest complications.

### 2.4. Outcomes

The primary outcome was complete wound closure, defined as full epithelialization without exudation. Wound closure was assessed by physicians during routine inpatient rounds and outpatient follow-up visits as part of standard clinical care. Assessment was primarily based on clinical examination and supported by serial clinical photographs. Photographs were not used as the sole basis for outcome evaluation. Outcome assessment was not restricted to the initial treating surgeon but was performed by different attending and resident physicians of the department. Secondary outcomes included time to wound closure, length of hospital stay, and the occurrence of wound-related complications. Time to complete wound closure was defined as the number of days from the day of initial surgical debridement (day 0) to complete epithelialization without exudation. As debridement and the first PLA matrix application were in all cases performed in the same procedure, day 0 corresponds to both the debridement and the first application. Length of hospital stay was recorded for all inpatients. Two patients were treated entirely in an outpatient setting due to individual clinical and organizational considerations. Complications were defined as wound-related events that delayed healing or required adaptation of the treatment strategy, such as infection, excessive exudation, allergic reaction, or wound dehiscence. Complications were categorized in minor or major. Major complications were defined as events requiring permanent cessation of the PLA matrix therapy, surgical revision, or systemic treatment. Minor complications included events requiring temporary interruption or modification of local wound care without permanent discontinuation of the planned therapy.

### 2.5. Data Analysis

All clinical data were retrospectively extracted via an internal data request. Data were exported from the hospital’s electronic medical records and manually transferred into a structured, anonymized spreadsheet using Microsoft Excel version 16.98 (Microsoft, Redmond, WA, USA). Quantitative variables were reported as means, medians, and standard deviations, while categorical variables were summarized using absolute and relative frequencies. Wound size was calculated as the product of wound length and width in square centimeters (cm^2^) based on clinical documentation. For wounds with irregular geometry, maximal dimensions were used to estimate surface area.

## 3. Results

In this study, 14 patients with a total of 16 chronic wound cases were treated using the novel polylactide matrix (see Table 1). In one patient, more than one anatomically distinct wound was evaluated. The mean age of the patients was 76.1 ± 10.2 (range, 62–93 years). Of the study population, seven patients were male and seven were female. Comorbidities were common, with diabetes mellitus and hypertension being the most frequent, each present in seven patients. Other common comorbidities included atrial fibrillation (*n* = 4) and heart insufficiency (*n* = 4).

The etiologies of the chronic wounds included ischemia in six cases, trauma in five cases, surgery-related wounds in three cases, and infection in two cases. The wounds were most commonly located on the lower leg (*n* = 8) and foot (*n* = 5), followed by the forearm (*n* = 2) and ankle (*n* = 1). The mean wound area was 23.6 ± 36.5 cm^2^. The median wound area was 8.0 cm^2^, with an interquartile range of 4.0–24.3 cm^2^.

A complete wound closure was achieved in 15 out of 16 cases, corresponding to a success rate of 93.8%. Twelve of these wounds (68.8%) achieved full epithelialization within 12 weeks. In three additional cases, wound closure occurred at a later stage (≥105 days). The mean length of hospital stay was 24.8 ± 8.1 days (median: 26 days; interquartile range: 25.5–26.0 days). Two patients were treated entirely on an outpatient basis; thus, no hospital stay was recorded for them. The mean time to complete wound closure was 72.9 ± 46.9 days, with a median of 60 days and an interquartile range of 45–91.5 days. Two cases were complicated by infection. Two wound-related complications occurred during the treatment period. Both events were classified as minor complications and consisted of local infections. In these two cases, the matrix was temporarily removed and local antimicrobial wound treatment was initiated. No systemic antibiotic therapy, surgical revision, or permanent cessation of therapy was required. No major complications were observed. In the only case in which complete wound healing was not achieved (therefore excluded from the time to wound closure analysis), the wound was infected with Pseudomonas aeruginosa. However, at the time of discontinuation of the polylactide matrix dressing, the wound was already nearly closed and further managed using antiseptic dressings. No cases of allergic reaction, wound dehiscence, or excessive exudation were observed.

The mean observational period was 79.6 ± 51.4 days, with a median routine follow-up time of 62.5 days (interquartile range: 45.0–106.5 days). Follow-up generally ended at complete wound closure and was not systematically continued thereafter.

**Figure 1:**
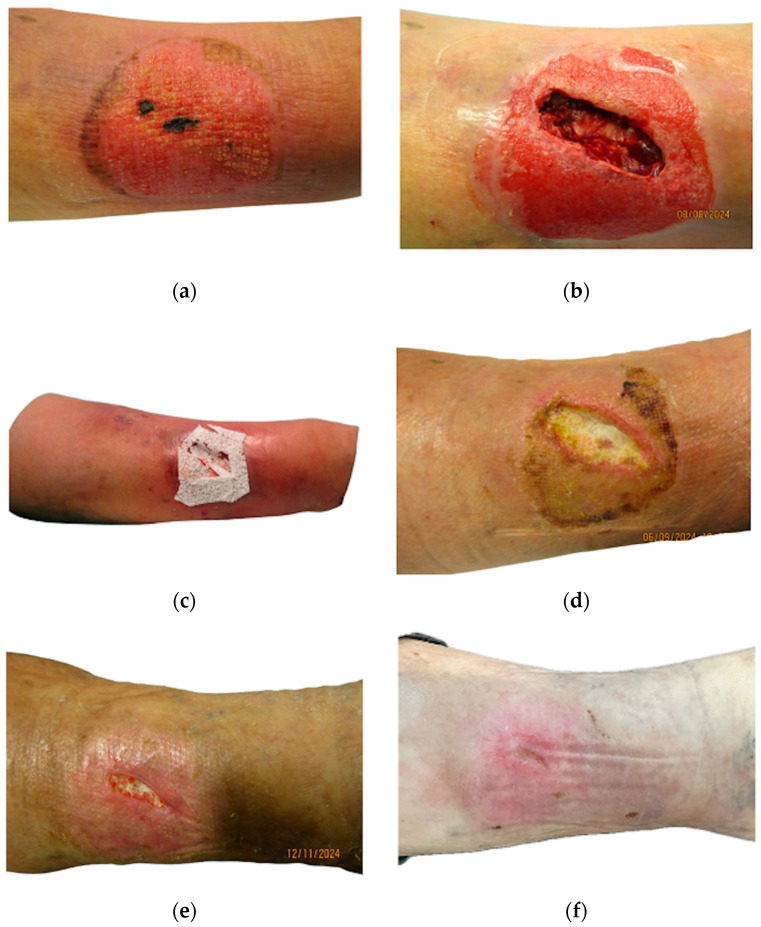
Clinical course of a lower leg wound following blunt trauma. The patient declined major surgical procedures due to comorbidities and explicitly requested the least invasive surgical approach possible. No negative pressure wound therapy (NPWT) applied. (**a**) (Day 0): Preoperative presentation showing an infected hematoma with overlying soft tissue necrosis. (**b**) (Day 0): Intraoperative findings reveal a soft tissue defect with exposed subcutaneous fat, crural fascia, and peritendineum. (**c**) (Day 0): Application of approximately 20 cm^2^ of bioresorbable polylactic acid matrix (PLA) into a 2.5 cm-deep defect. (**d**) (Day 29): Homogeneous granulation tissue with progressive resorption of the implanted matrix. No exudate issues; surrounding skin unremarkable. Silicone mesh used as secondary dressing. (**e**) (Day 96): Wound size reduced by over 70% with clean granulating wound bed. (**f**) (Day 205): Complete epithelialization and full wound closure.

**Figure 2:**
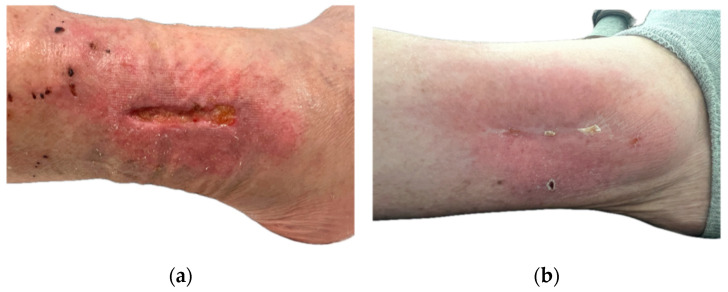
Chronic non-healing wound of the lower leg following orthopedic interventions. History of fibular osteosynthesis and subsequent implant removal at the lateral ankle joint due to local wound healing disorder. The patient declined major surgical revision and opted for a minimally invasive wound therapy. (**a**): Pre-treatment presentation of the chronic wound at the lateral lower leg with signs of local inflammation and impaired healing. (**b**): Post-treatment outcome showing complete wound closure. Healing time: 111 days.

Two representative cases are illustrated in Figure 1 and Figure 2, demonstrating the clinical course of chronic and hard-to-heal lower leg wounds treated with the bioresorbable polylactic acid matrix. Both patients provided written informed consent for the use of anonymized clinical photographs.

## 4. Discussion

The results of this study demonstrate that the use of the polylactide matrix as part of a structured multimodal wound care approach was associated with a high overall rate of complete wound closure (93.8%) across a heterogeneous cohort of patients with diverse comorbidities and wound etiologies. These findings indicate that the matrix may represent a promising adjunctive treatment option for complex and therapy-resistant wounds, particularly in elderly and multimorbid patients who are frequently encountered in clinical practice and who may not be suitable candidates for more extensive surgical procedures such as flap surgery.

At the same time, the interpretation of these findings should consider the retrospective and uncontrolled study design, which does not allow for definitive causal statements regarding comparative effectiveness. The favorable clinical outcomes observed are likely the result of the combined effect of prerequisite surgical debridement, comprehensive multimodal wound management, and adjunctive matrix application.

In comparison to the randomized controlled trial by Liden et al., which evaluated the same PLA matrix in Wagner grade 1 and 2 diabetic foot ulcers, our study demonstrates similar healing times and ultimately a higher rate of complete wound closure. While Liden et al. reported a median closure time of approximately 65 days and a healing rate of 80% at 12 weeks, we observed an overall closure rate of 93.8% during extended follow-up, with a mean time to healing of 72.9 days [14]. Notably, our cohort included a broader range of wound etiologies—such as ischemic, traumatic, and infected wounds—reflecting more heterogeneous and clinically complex conditions that are representative of real-world patient populations typically seen in wound care clinics [15].

Compared to other regenerative wound therapies, such as the autologous blood clot matrix described by Kushnir et al., which achieved complete healing in 78% of chronic wounds within a treatment period of up to 61 days, the average healing time observed in our study (73 days) appears clinically comparable—despite the inclusion of more complex cases, such as infected and ischemic wounds, most of which presented with significantly greater wound depth [16]. Notably, the remarkably high rate of complete epithelialization (93.8%) achieved with the polylactic acid matrix underscores its strong regenerative potential, even under unfavorable local and systemic conditions [16].

Given the wide range of currently available, often high-cost skin substitutes and soft tissue replacements, a direct one-to-one comparison is desirable but remains challenging due to the highly heterogeneous nature of wound conditions. The study by Düppers et al., for example, demonstrates that intact Fish Skin Grafts (iFSGs) can significantly contribute to granulation tissue formation, even in extreme post-necrotizing fasciitis defects—albeit as part of a multi-stage treatment concept [17]. In contrast, no such multi-stage approach was necessary in our study, as complete wound healing was achieved solely through the use of the polylactide matrix. Skin grafts were not required in any case in our study.

The most extensive study to date comparing polylactide-based wound matrices with collagen wound dressings and intact fish skin grafts (iFSGs) is the multicenter retrospective analysis by Liden et al., which evaluated 131 chronic wounds, including diabetic foot ulcers and venous leg ulcers [15]. In comparison to the PLA cohort, our results demonstrate a similar time to healing and an even higher rate of complete wound closure (93.8% vs. 86%). Our outcomes appear favorable when viewed in the context of the reported healing rates and time to closure in the iFSG cohort (healing rate 62%, median time to closure: 12 weeks) and the collagen group (healing rate 31%, closure after 16–17 weeks). However, substantial differences in study design, patient populations, wound characteristics, and outcome definitions limit direct comparability [18].

Structured wound evaluation and algorithm-based treatment concepts are increasingly recognized as essential components of modern chronic wound management. Standardized assessment frameworks aim to improve clinical decision-making, enhance reproducibility, and facilitate timely adaptation of therapeutic strategies, independent of the specific wound dressing applied. In this context, Cunha et al. described algorithm-driven approaches for systematic wound evaluation and treatment selection and demonstrated the potential of computational and mobile technology-based systems to support objective wound documentation and longitudinal monitoring [19,20].

The growing importance of technology-assisted wound diagnostics is further highlighted by recent reviews on imaging-based approaches in wound medicine. Li et al. demonstrated that advanced imaging and camera-based techniques—including optical, spectral, and thermal imaging—can substantially contribute to objective assessment of wound status, reduce interobserver variability, and support longitudinal wound evaluation in both inpatient and outpatient settings. Such technologies further enable a more dynamic characterization of the wound microenvironment beyond purely morphological parameters [21]. In this context, standardized and technology-assisted wound assessment approaches represent a promising avenue for future investigations aimed at improving the precision and reproducibility of wound healing evaluation.

A key aspect in managing chronic wounds is their considerable economic burden on the healthcare system. A German study including 502 patients with a mean age of 71 years and an average wound duration of 9 years reported mean annual total costs of €9060 per patient [18]. Against this background, the use of a synthetic polylactide matrix offers potentially significant cost-saving advantages. On the one hand, direct costs can be reduced due to the matrix’s high efficacy, which may minimize the need for frequent dressing changes, additional antimicrobial wound dressings, or surgical interventions such as skin grafting. On the other hand, indirect cost savings may result from shorter hospital stays, reduced nursing effort, and a faster transition of patients to outpatient care.

### 4.1. Strengths of the Study

One of the main strengths of this study lies in its real-world clinical setting: all patients were treated as part of routine care at a single center in Berlin, ensuring high external validity for everyday clinical practice. Importantly, with the exception of the predefined adult age criterion, the study included all consecutive patients treated with the polylactide matrix during the study period, minimizing selection bias. This approach minimizes selection bias and reflects a truly heterogeneous patient population. Furthermore, the study covers a broad spectrum of wound etiologies, including ischemic, infected, traumatic, and postoperative chronic wounds—defined as wounds that fail to show significant healing within four weeks despite appropriate treatment—highlighting the versatility and applicability of the synthetic matrix across diverse clinical scenarios. The diversity of clinical presentations allowed treatment decisions to be adapted to individual wound characteristics and patient-specific constraints, illustrating the practical applicability of the matrix within a personalized therapeutic approach.

### 4.2. Limitations of the Study

This study has several limitations that should be acknowledged. First, its retrospective design without a control group limits the ability to draw causal conclusions or to directly compare the effectiveness of the polylactide matrix with standard wound care or other advanced wound care products. Second, the relatively small sample size precludes statistically robust subgroup analyses; therefore, the results should be regarded as descriptive and exploratory. Third, although all patients treated with the PLA Matrix were included, selection bias cannot be ruled out because wounds that were considered complicated may have been treated with a more established therapy.

Fourth, although potential economic benefits were discussed based on clinical outcomes such as wound closure rates and hospitalization duration, no formal health economic evaluation was performed, and cost-effectiveness was not quantitatively assessed. Finally, the absence of a standardized application protocol for the matrix reflects real-world clinical practice but may have led to differences in treatment strategies and follow-up intervals. Follow-up was therefore limited to wound closure, as this reflects the usual clinical reality. Long-term outcomes such as recurrence rates and improvement in quality of life after treatment were not evaluated. This would require a standardized follow-up and patient satisfaction questionnaires.

## 5. Conclusions

Our findings demonstrate that the polylactic acid matrix achieved a high rate of short-term wound closure in a heterogeneous cohort of patients with chronic wounds, with a mean healing time of 73 days and without the need for additional skin grafting or further surgical interventions. Although the results of our study indicate promising clinical efficacy, the standard follow-up care provided to patients does not allow any conclusions to be drawn about long-term results. This would require standardized follow-up examinations over a period of at least 3–6 months, extending beyond the time of wound healing. From an economic perspective, this treatment approach may offer significant cost savings by reducing the length of hospital stays, lowering material consumption, and eliminating the need for secondary interventions. Future prospective, multicenter studies with integrated cost analyses are warranted to validate these promising results further.

## Figures and Tables

**Table 1:** Patient characteristics, wound etiology, location, treatment course, and outcomes in all chronic wound cases treated with polylactic acid matrix.

Age [Years]	Sex [M/F/D]	Comorbidities	Wound Etiology	Location	Size [cm × cm]	Area [cm^2^]	Length of Hospital Stay [days]	Full Wound Closure Achieved [Yes/No]	Time to Complete Wound Closure [days]	Occurring Complications During Treatment Period
67	M	AF, DM, HI, HTN	Ischemic	Lower Leg	2 × 2	4.0	15	Yes	65	
			Ischemic	Ankle	2.5 × 2	5.0	36	Yes	44	
			Ischemic	Foot	4 × 3	12.0	34	Yes	105	
62	M	PAD/CVI	Infectious	Foot	5 × 5	25.0	26	No	n/a (not closed)	Local Infection
83	M	DM	Post-Traumatic	Forearm	15 × 8	120.0	25	Yes	115	
60	M	DM	Post-Traumatic	Lower Leg	2 × 2	4.0		Yes	50	
77	F	COPD, DM, HTN	Post-Traumatic	Lower Leg	6 × 4	24.0	35	Yes	205	
93	F	AF, HTN, Obesity, PAD/CVI	Iatrogenic/Postoperative	Foot	2 × 2	4.0	26	Yes	78	
84	F	AF	Ischemic	Lower Leg	7 × 5	35.0		Yes	45	
83	F	AF, DM, HTN	Post-Traumatic	Foot	2 × 2	4.0	26	Yes	74	
86	F		Infectious	Lower Leg	2 × 2	4.0	26	Yes	60	
79	F	Cancer, HI, HTN	Iatrogenic/Postoperative	Lower Leg	4 × 2	8.0	15	Yes	45	
83	M	HI	Iatrogenic/Postoperative	Lower Leg	4 × 2	8.0	26	Yes	111	
71	F	COPD, DM, HI	Ischemic	Lower Leg	3 × 3	9.0	26	Yes	23	Local Infection
74	M	HTN	Traumatic	Forearm	12 × 9	108.0	25	Yes	16	
63	M	DM, HTN, liver cirrhosis	Ischemic	Foot	2 × 2	4.0	26	Yes	58	

Abbreviations: AF, atrial fibrillation; DM, diabetes mellitus type 2; HI, heart insufficiency; HTN, arterial hypertension; PAD, peripheral arterial disease; CVI, chronic venous insufficiency; COPD, chronic obstructive pulmonary disease.

## Data Availability

The original contributions presented in this study are included in the article. Further inquiries can be directed to the corresponding author.
